# Supplementary material for: Effects of Different-Syllable Aggressive Calls on Food Intake and Gene Expression in Vespertilio sinensis
Source: Animals (Basel). 2023 Jan 15;13(2):306. doi: 10.3390/ani13020306 (PMC9854892; doi:10.3390/ani13020306)
Supplement: Supplementary file 1 [file animals-13-00306-s001.zip › animals-2063478-supplementary.pdf]

Supplementary Information for

Effects of different-syllable aggressive calls on food intake and gene expression in *Vespertilio sinensis*

Xin Li, Ruizhu Zhou, Lei Feng, Hui Wang, Jiang Feng, Hui Wu

Details of methods of RNA extraction, sequence splicing, and annotation

RNA extraction

Total RNA was extracted from the kidney of bats according the manufacturer's instructions (Invitrogen, Carlsbard, CA, USA) and genomic DNA was removed using DNase I (TaKara). Then, the integrity and purity of the total RNA quality was determined by 2100 Bioanalyser (Agilent Technologies, Inc., Santa Clara CA, USA) and quantified using the ND-2000 (NanoDrop Thermo Scientific, Wilmington, DE, USA). Only high-quality RNA sample (OD 260/280 = 1.8 to 2.2, OD 260/230 ≥ 2.0, RIN ≥ 8.0) was used to construct sequencing library.

Library preparation and Illumina Hiseq xten/NovaSeq 6000 Sequencing

To construct a general reference transcriptome for subsequent analysis, the cDNA library sequences of 12 brain tissues sampled at the same time point and the kidney tissue sequences were put together for splicing and assembly. The specific brain data analysis is published elsewhere. RNA purification, reverse transcription, library construction, and sequencing were performed at Shanghai Majorbio Bio-pharm Biotechnology Co., Ltd. (Shanghai, China) according to the manufacturer's instructions (Illumina, San Diego, CA). The kidney and brain tissues RNA-seq transcriptome libraries were prepared using Illumina TruSeqTM RNA sample preparation Kit (San Diego, CA). Poly (A) mRNA was purified from total RNA using oligo-dT-attached magnetic beads and then fragmented by fragmentation buffer. Taking these short fragments as templates, double-stranded cDNA was synthesized using a Superscript double-stranded cDNA synthesis kit (Invitrogen, CA) with random hexamer primers (Illumina). Then, the synthesized cDNA was subjected to end-repair, phosphorylation, and 'A' base addition according to Illumina's library construction protocol. Libraries were size-selected for cDNA target fragments of 200 to 300 bp on 2% Low Range Ultra Agarose followed by PCR amplified using Phusion DNA polymerase (New England Biolabs, Boston, MA) for 15 PCR cycles. After quantified by TBS380, two RNAseq libraries were sequenced in single lane on an Illumina Hiseq xten/NovaSeq 6000 sequencer (Illumina, San Diego, CA) for 2 × 150 bp paired-end reads.

De novo Assembly and Annotation

The raw paired-end reads were trimmed and quality controlled by SeqPrep (https://github.com/jstjohn/SeqPrep) and Sickle (https://github.com/najoshi/sickle) with default parameters. Then, clean data from the samples were used to perform de novo assembly with Trinity (http://trinityrnaseq.sourceforge.net/) (Grabherr M. G et al., 2011). All the assembled transcripts were searched against the NCBI protein Non-redundant (NR), Cluster of Orthologous Groups (COG), and Kyoto Encyclopedia of Genes and Genomes (KEGG) databases using BLASTX to identify the proteins that had the highest sequence similarity with the given transcripts to retrieve their function annotations and a typical cut-off E-values less than 1.0×10^{-5} was set. BLAST2GO (http://www.blast2go.com/b2ghome) program was used to get GO annotations of unique assembled transcripts for describing biological processes, molecular functions, and cellular components (Conesa A et al., 2005). Metabolic pathway analysis was performed using the Kyoto Encyclopedia of Genes and Genomes (KEGG, http://www.genome.jp/kegg/) (Mkas, 2000).

Table S1. Sequencing and assembly statistics of kidney samples for *V. sinensis*.

	**Group A**	**Group B**	**Group C**	**Group D**
**Sequencing**				
Total Sequences （bp）	24,664,998,360	25,108,393,250	22,972,906,252	21,023,654,802
Total reads (raw reads)	163,344,360	166,280,750	152,138,452	139,229,502
Clean reads	161,685,102	164,491,068	149,810,472	137,625,012
Ratio of clean/raw	98.98%	98.92%	98.47%	98.85%
**Assembly**				
Unigenes	195,827			
N50	1,979			
N90	3,491			
Max. length	24,819			
Min. length	201			
Ave. length	1,151			

Table S2. Summary of the kidney samples transcriptome annotation.

Database	Number of gene	Percentage (%)
GO	30665	24.81
KEGG	18318	14.82
eggNOG	26158	21.17
NR	40527	32.79
Swiss-Prot	26272	21.26
Pfam	20137	16.29
At least one database	42577	34.45
